# You can have both: Coaching to promote clinical competency and professional identity formation

**DOI:** 10.1007/s40037-020-00612-1

**Published:** 2020-08-17

**Authors:** Andrew S. Parsons, Rachel H. Kon, Margaret Plews-Ogan, Maryellen E. Gusic

**Affiliations:** 1 Department of Medicine, University of Virginia School of Medicine, 1215 Lee St., 22908-0422 Charlottesville, VA USA; 2 Department of Public Health Sciences, University of Virginia School of Medicine, Charlottesville, VA USA; 3 University of Virginia School of Medicine, Charlottesville, VA USA

**Keywords:** Coaching, Clinical skills, Professional identity formation, Assessment, Trust, Longitudinal relationship, Self-regulated learning

## Abstract

Coaching is a critical tool to guide student development of clinical competency and formation of professional identity in medicine, two inextricably linked concepts. Because progress toward clinical competence is linked to thinking, acting and feeling like a physician, a coach’s knowledge about a learner’s development of clinical skills is essential to promoting the learner’s professional identity formation. A longitudinal coaching program provides a foundation for the formation of coach-learner relationships built on trust. Trusting relationships can moderate the risk and vulnerability inherent in a hierarchical medical education system and allow coaching conversations to focus on the promotion of self-regulated learning and fostering skills for life-long learning. Herein, we describe a comprehensive, longitudinal clinical coaching program for medical students designed to support learners’ professional identify formation and effectively promote their emerging competence.

## Introduction

Coaching, while common in business and sports, has only gained considerable traction in medical education over the last five years [1, 2]. Within undergraduate medical education, coaching programs are most commonly organized to address one of three domains: professional identity formation, professionalism or academic performance [3]. In the vast majority of programs, coaches do not formally assess students in any domain and rarely directly observe clinical performance [3]. Sawatsky et al. recently described a potential conflict between the development of professional identity and competency-based programs of assessment, highlighting the challenge learners perceive in exposing vulnerability to those who assess their performance [4]. Though the ideal competency-based program of assessment [5] includes regular low-stakes assessments, these authors state that learners “tend to view all observation as assessment and suspect that any and all aspects of their performance have implications for their permanent record and even their training or career advancement” [4]. But, how can coaches facilitate identity formation and socialization into the physician community of practice without being able to foster habits of continuous performance improvement?

In this paper, we describe a comprehensive clinical coaching program implemented to support medical students’ professional identify formation and effectively promote their emerging competence. Instead of “either/or”, we consider these goals of our coaching program to be interdependent. Applying the principles of polarity thinking^TM^, we have used a “both/and” approach to design our program [6]. We begin by distinguishing coaching from other supervisory roles in medical education and argue that coaching is a critical tool to guide student development of clinical competency and professional identity. We conclude by delineating the structure of our coaching program, which we hope can serve as a model for other institutions planning curricula that incorporate coaching. We highlight how within a trusting relationship, medical educators can explicitly use coaching conversations as a tool to advance clinical competency while concurrently facilitating professional identify formation in their learners.

## Longitudinal coaching to build trust

Deiorio et al. define the process of coaching in medical education as “facilitating learners achieving their fullest potential” [6]. A coach evaluates learner performance by reviewing data from various assessment methods (e.g., multiple choice exams, essay examinations, observed structured clinical examinations, direct observation in the workplace [7]) in order to identify areas for development and, while helping to create an action plan, also promoting accountability in the learner to meet their needs [1]. This definition distinguishes coaching from more traditional relationships in medical education, specifically advising and mentoring. An advisor “tells”; offering strategies to apply to a specific activity, such as a career decision, while a mentor “shows the way”; typically engaging in a long-term relationship with a mentee during which a broad range of topics [8] are addressed. The wise and effective coach “asks questions”; promoting self-monitoring and learner-centered skill development as part of a longitudinal relationship [8].

Trust, defined as the “belief that someone is reliable, good, honest, effective,” [9] is essential to counter the potential risk and vulnerability related to the hierarchical nature of the teacher-learner relationship [10]. Across the continuum of medical education, the feedback literature suggests that learners incorporate feedback best when a longitudinal relationship is formed with a trusted person and an “educational alliance” exists [11–14]. A pertinent example, the R2C2 feedback model, has been shown to be effective in fostering learner self-reflection and self-direction in addition to facilitating collaborative development of a plan to change behavior [15]. This model, introduced for use in graduate medical education, consists of four phases: relationship building, exploring reactions to feedback, exploring understanding of feedback content, and coaching for performance change [16].

Most medical education learners, however, have limited longitudinal relationships within clinical environments [17]. Within the clerkship environment, students describe appropriate trust when supervisors provide “opportunities for coaching, feedback, and scaffolding of their learning [18].” Yet, clinical teachers rarely observe learners with patients [15] and are commonly asked to assess a learner’s ability with limited knowledge of the learner or the learning environment [10]. A longitudinal coaching program can promote formation of coach-learner relationships built on trust [19]. Trust is reinforced when the role of the coach is clearly defined and learners and coaches have shared expectations for the goals of the relationship [20, 21]. Mutual engagement, confidential conversations, and trust are required so that coaches are able to help a learner reflect, and have insight into where they are on the continuum of clinical and professional development, and how to move forward. These are elements that exist in a longitudinal relationship in a way that may be rarely seen in brief teacher-learner interactions [21].

## Coaching to promote clinical competency

Traditional medical education occurs in distinct phases as students focus on biomedical systems early in their schooling and then transition to the clinical environment. The organization of curricula can feel disjointed for learners as they transition between different courses with numerous teachers over varying periods of time [22]. The introduction of clinical learning early and in all phases of curricula is an effort to better integrate content, advance learning [23], and enhance clinical skill development. Each phase of the curriculum may present unique challenges requiring adaptive learning strategies [24, 25]. Faced with these challenges, learners can accelerate adaptation and innovation to meet identified learning needs using self-regulated learning skills [26]. Self-regulated learning is a process by which learners set informed goals, engage in the learning process, and then evaluate learning through self-reflection and self-evaluation to inform future goal development [27].

Once coaches and learners have forged an alliance built on trust, coaching conversations can serve as a tool to encourage self-regulated learning [28, 29] and establish skills for life-long learning [30–32], both of which are necessary to promote clinical skill development. Coach-learner meetings offer a structured opportunity for students to discuss personalized, actionable goals and action plans as they progress through different phases of medical school [33]. Coaches can enhance development of clinical skills by informing each phase of the self-regulated learning process specific to clinical competency, the ability to do the things a physician is supposed to do.

## Coaching to foster professional identity formation

Professional identity formation is a developmental process whereby the characteristics of the medical profession are internalized by medical students [34, 35]. Even experienced clinical teachers can struggle to describe their influence on learners’ professional identity. Once prompted to focus on their career path, these teachers describe caring for patients as integral to forming their professional identity [36]. Just as early clinical exposure promotes clinical skill development, a longitudinal relationship with a patient, beginning in their first year of medical school and overseen by a coach, can establish a critical three-way trusting relationship between student, coach and patient that can be an important focal point for the student’s learning and growth as a professional.

Cruess et al. describe the goal of identity formation as engaging learners as active participants in the process and reflecting on their progress toward “thinking, acting, and feeling like a physician” [35]. Coaches can encourage learners as they internalize norms and values and, at the same time, challenge learners to reflect on their experiences with the medical profession. Students enter medical school with unique expectations of what it means to be a physician [37, 38]. They then undergo a socialization process shaped by their previous experiences and the clinical curriculum, most prominently during clerkship learning experiences [39]. A coach’s knowledge about their learners’ clinical experiences and developing skills is essential to foster learners’ professional identity formation. Feedback about progress towards the goal of becoming a physician facilitates identity formation [34] and coaching to promote a learner’s reflection on competency and on their emerging identity is complementary.

## Both/And, an integrated coaching program

At the University of Virginia School of Medicine, we have implemented a comprehensive clinical coaching program, with dedicated physician coaches who work longitudinally with students, and through reflective dialogue guide students to explore their emerging identity as physicians and to create meaningful learning plans for learning using data from clinical assessments. We describe the structure of our coaching program by outlining the expectations of the role with respect to instruction and coaching in each phase of the curriculum (Tab. 1). We then explain the importance of nurturing a professional culture of coaching [40] supported by infrastructure that creates a sense of community for coaches [41], the importance of institutional support, and the role of feedback and evaluation for coaches in the program.

**Table 1:** Components of our comprehensive coaching program to promote clinical competency and professional identity formation: Structure, representative curricular topics, and illustrative educational methods

Phase of the curriculum		Curricular topics	Educational methods
*Pre-clerkship (18 months):* One faculty coach and six students meet as small group weekly for four hours	*Clinical competency*	– History taking – Physical exam skills – Communication skills – Clinical reasoning – High value care – Social influences on health	– Case-based role plays – Simulation with standardized patients and high fidelity simulators – Direct observation with coach and peer feedback – EPA assessments by faculty (not coaches) – Review of clinical assessment data with coaches – Narrative medicine – House call, narrative interview of student’s patients – Reflection on student doctor relationship – Individualized formative feedback and goal setting
	*Professional identity formation*	– Authentic student doctor role – Relational skills – Professional boundaries – Physician well-being – Positive practices	
*Clerkship (12 months) and Post-clerkship (14 months):* One faculty coach and six students meet as small group for two hours quarterly; individual coach-learner meetings quarterly	*Clinical competency*	– Communication skills – Patient care skills – Clinical reasoning skills	– Reflection on, and review of clinical assessment data as a tool for learning – Co-construction of learning goals for ongoing development (clinical competency and professional identity formation) by coaches and students – Reflective writing – Facilitated debriefing of critical incidents – Small group discussion to reflect on student-doctor relationship – Individualized formative feedback and goal setting
	*Professional identity formation*	– Developmental progression/performance expectations related to graduated autonomy – Experience/impact of critical incidents – Evolution of student-doctor relationship	

## Coaching in the pre-clerkship curriculum

In the first month of the curriculum, students are matched with a group of five or six other students, a coach who will work with them across all phases of the curriculum, and for each student, a patient who they will follow longitudinally. As part of the 18-month pre-clerkship curriculum, coaches meet with students weekly in small groups to teach and promote clinical skill development and professional identity formation. Week one discussions center on physician wellbeing and identifying characteristics of the exemplary, good, and wise doctor [42]. With coaches as their guides, students engage in positive practices like mindfulness. Group sessions begin with an appreciative check in to allow the coach and all of the learners to build trust, to approach conversations with a sense of curiosity and to seek the “goodness” in one another. Students are asked, “What has gone well this week?” Medical students traditionally are surrounded by judgment, often internalizing this perception to the point that they are uncomfortable acknowledging shortcomings. Moving judgment to curiosity teaches students to approach their own and others’ mistakes as an opportunity to ask the curious question “I wonder how…” Coaches create a safe environment and model behaviors as they discuss their own clinical experiences. As coaches tell stories about times during which they were uncertain, didn’t have the answer, or questioned their own performance, they allow learners to see that seasoned clinicians also experience vulnerability. Coaches’ willingness to share challenges and fallibilities allows students to see the benefit of reflection and honest self-assessment and is an important way in which trust is established in the coach-learner relationship [43].

With these concepts as a foundation, students learn history taking, physical exam, and clinical reasoning skills using case-based role-play exercises, simulation, and practice with standardized patients. In facilitated discussions, students reflect on social issues in medicine, practice cost-conscious decision-making, and are introduced to advanced communication techniques such as breaking bad news. Coaches do not complete clinical assessments; rather they regularly observe students during these activities and provide structured formative feedback. Following summative observed structured clinical examinations, coaches watch recordings with their students to identify strengths and areas in need of improvement. At the end of each semester, coaches meet one-on-one with each student to reflect, provide feedback, and help the student set goals for future development.

The longitudinal patient relationship program begins in the first semester of medical school. This patient program was first piloted with a small group of 12 students [42] and two faculty mentors, and expanded over the ensuing four years to include 36 students and six mentors per year. The pilot allowed us to test the feasibility of fully integrating this component with the coaching program for a larger number of students, patients and faculty, to transition the faculty role from mentor to coach, and to determine the amount of time needed for faculty to successfully fulfill the role of coach for four groups of medical students simultaneously (each coach working with one group of six students from each medical school class). Students begin the relationship by getting to know their patients as persons, through a narrative interview and house call. Coaches accompany students on visits to the patient’s home. This activity takes place on the patient’s “turf”, and although it may conjure up a sense of vulnerability, builds trust as the pair shares the experience together. Initial interactions are also critical to establish trust that the patients quickly begin to rely on, while the students develop a sense of responsibility toward their patients. The students attend clinic visits, see their patients when hospitalized, and do additional house calls when appropriate. They go through crises with their patients and the patient’s family including illness and in some cases, a patient’s death. Over time, the student-patient relationship grows and changes. As students accumulate medical knowledge and clinical skills, propelling their transition from layperson to clinician, their role within the student-patient relationship undergoes a similar transition from friend to healthcare advocate. Faculty coaches oversee this developing relationship, help the students manage any challenges that arise, and debrief assignments such as working with their patients on medication reconciliation or motivational interviewing to help their patients identify and achieve a behavior change goal. These activities give students a meaningful role, solidify clinical skills and are designed to highlight critical issues related to identity formation.

When confronted with the educational challenges posed by the COVID-19 pandemic, our coaches effectively shifted to interacting with students in their coaching groups using a web-based platform. Coaches maintained their focus on promoting maturation of clinical skills by adapting teaching strategies to engage learners during the virtual sessions. For example, coaches had students demonstrate physical exam maneuvers on roommates, family members, or an inanimate object like a doll. Small group discussions addressed emerging topics related to the new ways physicians are engaging in care and students shared stories about how their patients were experiencing social isolation during the crisis. In an unprecedented time, coaches emphasized the uniqueness of this moment as an unforgettable step in the formation of each learner’s professional identity.

## Coaching in the clerkship and post-clerkship phase of the curriculum

Coaches meet with students in groups and individually on a quarterly basis to promote professional identity formation and clinical competency. The quarterly group meetings focus on a critical incident [44] topic related to professional identify formation. Examples include engaging students in reflection and discussion of challenges they are struggling with related to their experiences in a clinical situation. Generally the group meetings begin with reflective writing using a prompt about a topic. For example: “Your clinical year is full of first times….first birth, first death, first mistake, first time someone called you their student doctor. Write about a first time you experienced over the past few months.” The challenges are discussed as formative experiences. Later in the clerkship year students are asked to “write about a time when you were involved in or witnessed a medical error. How was it handled in the aftermath? Was there a debriefing with you or your team? What helped you in the aftermath of that event?”

Coaches meet quarterly one-on-one with their students throughout the clerkship phase. In preparation for individual meetings, students and faculty coaches review data from clinical assessments including the results of Entrustable Professional Activity (EPA) assessments [45], clinical evaluations from clerkship rotations and reports from the Entrustment Committee. The Entrustment Committee is charged with aggregating data from EPA assessments to determine students’ readiness to engage in advanced clinical electives in the post-clerkship phase of the curriculum [46]. Using this data and self-reflection on performance during clerkships, students prepare individualized learning plans [47, 48]. Coaches also review the data and complete their assigned section of the learning plan, reflecting on students’ self-assessment and identifying additional considerations to be discussed at the coaching meetings. At the coaching meetings, coaches and students co-create [49] a finalized list of learning goals and co-construct an action plan to meet these goals. Subsequent meetings assess students’ progress towards achieving their learning goals, assess their emerging competence as documented in the results of clinical assessments, and develop additional goals to promote ongoing development. This process intentionally mirrors the metacognitive and strategic thinking components of self-regulated learning so that students can practice and apply these skills and be motivated to incorporate these behaviors to achieve continuous, lifelong improvement as physicians.

Coaches continue to meet with students in groups on a quarterly basis during the post-clerkship phase of the curriculum. Sessions include a general “check-in” followed by positive practice exercises to promote wellness. Coaches guide student reflection on recent clinical experiences by asking questions such as: “What were the important features of this experience and how does it influence the choices you are making about your career?” Students complete writing assignments to promote reflection on a moment when they felt like the doctor they want to be. The partnership between coach and student during the clinical phases of the curriculum is structured intentionally to address both clinical competency and professional identity formation as critical aspects of being a physician.

## Cultivating a coaching culture

Successful implementation and maintenance of our coaching program requires deliberate, ongoing cultivation of a professional culture and sense of community among coaches. Monthly faculty development sessions and quarterly large group retreats ensure coaches similarly actualize the expectations of the role and focus coaching conversations on learners’ growth and development. [40]. These sessions center on improving skills in coaching, teaching, helping struggling students, and in understanding and promoting the process of professional identity formation. The retreats are attended by coaches, student affairs deans, school of medicine leadership, program leaders, and topical experts (e.g. self-regulated learning, motivational interviewing, evidence-based physical examination). Small group “brown-bag” sessions are held twice monthly to ensure an ongoing dialogue between coaches and to allow them to share effective coaching techniques with their peers. Coaches, each with substantial experience in medical education, are asked to take on faculty development roles for their peers that fit with their interest and expertise. Coaches also participate in key events initiating students into the profession of medicine including the white coat ceremony, the student clinician’s ceremony, Match Day and graduation. Finally, the coaching small groups are organized within an overarching system of student support. Six coaching groups are aligned with one of our four colleges; each led by a student affairs dean. This structure provides a well-integrated system for student support; coaches support learners’ clinical development and professional identify formation while the college deans work longitudinally to support students’ overall academic success.

## Institutional support for the coaching program

Investment from the School of Medicine has been vital for the implementation and subsequent success of the coaching program. Key to the implementation of the program was the development of a cadre of dedicated coaches who were able to dedicate a significant part (30%) of their FTE to this professional role. It was critical to specifically delineate responsibilities and activities associated with the role not only to ensure clarity for coaches and learners and ensure that faculty have a coaching mindset in their work with learners [46] but also to be able to calculate the percent of professional time necessary to fulfill all of the expectations of the program. Through an application, interview and selection process we were able to create a cadre of highly dedicated career educators. This group of faculty are highly engaged in continuous professional development and improvement to enhance their own performance and the program as a whole.

## Feedback and evaluation in the coaching program

Program leaders meet with each coach on an annual basis and review students’ evaluation reports related to their coach’s performance. Review meetings focus on what is going well for an individual coach, challenges they have encountered, discussions around content to ensure standardization of teaching and approach to coaching conversations, and how program leaders can provide greater support. In addition, as a critical component of our curriculum, the coaching program is reviewed annually as a part of program evaluation overseen by the School of Medicine.

## Conclusion

Coaching, a relatively new concept in academic medicine, provides a tool to promote both emerging clinical competency and professional identity formation among medical students. Explicit strategies used within longitudinal coaching relationships built on trust can help learners use data from assessments for continued development and learn what it “is” to be a physician as they navigate challenges in the clinical environment. Coaching conversations, when structured to foster learners’ self-assessment and accountability, identification of needs, and co-creation of action plans, can advance skills essential to physician-hood. Coaches’ engagement with learners in this way and over time guides the development of self-regulated learning skills and lays the foundation for lifelong reflective practice as a physician.

We conclude by sharing lessons learned from the implementation of our coaching program. Clear definition of the role of the coaches and the use of a coaching approach was an intentional decision to ensure alignment with the interdependent goals of the program. Institutional support for coaches to dedicate a designated proportion of professional effort and integration of this program with the existing system of student support provided the foundation for the mutual engagement of coaches and learners. In implementing the program, the importance of ongoing professional development, feedback and evaluation of the coaches and the program was highlighted. Professional development addressed the need to build coaches’ skills, continuously reinforce the goals and tenets of the program and strengthen the professional culture of coaching. We hope our robust clinical coaching program structured around longitudinal coach-student partnerships, student-patient relationships, and multimodal faculty development, serves as a model to medical educators and institutions designing similar programs.

## References

[1] Lovell B (2018). What do we know about coaching in medical education? A literature review. Med Educ.

[2] Watling CJ, Ginsburg S (2019). Assessment, feedback and the alchemy of learning. Med Educ.

[3] Wolff M, Hammoud M, Santen S, Deiorio N, Fix M (2020). Coaching in undergraduate medical education: a national survey. Med Educ Online.

[4] Sawatsky AP, Huffman BM, Hafferty FW. Coaching versus competency to facilitate professional identity formation. 2020. https://journals.lww.com/academicmedicine/Abstract/publishahead/Coaching_Versus_Competency_to_Facilitate.97339.aspx. Accessed 12 Apr 2020, Acad Med. 10.1097/ACM.0000000000003144 31895702

[5] Schut S, Driessen E, van Tartwijk J, van der Vleuten C, Heeneman S (2018). Stakes in the eye of the beholder: an international study of learners’ perceptions within programmatic assessment. Med Educ.

[6] Deiorio NM, Carney PA, Kahl LE, Bonura EM, Juve AM. Coaching: a new model for academic and career achievement. 2016. https://www.ncbi.nlm.nih.gov/pmc/articles/PMC5136126/. Accessed 12 Apr 2020, Med Educ Online. 10.3402/meo.v21.33480 PMC5136126 27914193

[7] Lockyer J, Carraccio C, Chan M-K (2017). Core principles of assessment in competency-based medical education. Med Teach.

[8] Marcdante K, Simpson D (2018). Choosing when to advise, coach, or Mentor. J Grad Med Educ.

[9] Merriam-Webster. Definition of trust. 2020. https://www.merriam-webster.com/dictionary/trust. Accessed 14 Apr 2020.

[10] Abruzzo D, Sklar DP, McMahon GT (2019). Improving trust between learners and teachers in medicine. Acad Med.

[11] Telio S, Ajjawi R, Regehr G (2015). The “educational alliance” as a framework for reconceptualizing feedback in medical education. Acad Med.

[12] Harrison CJ, Könings KD, Dannefer EF, Schuwirth LWT, Wass V, van der Vleuten CPM (2016). Factors influencing students’ receptivity to formative feedback emerging from different assessment cultures. Perspect Med Educ.

[13] Mann K, van der Vleuten C, Eva K (2011). Tensions in informed self-assessment: how the desire for feedback and reticence to collect and use it can conflict. Acad Med.

[14] Sargeant J (2019). Future research in feedback: how to use feedback and coaching conversations in a way that supports development of the individual as a self-directed learner and resilient professional. Acad Med.

[15] Sargeant J, Lockyer JM, Mann K (2018). The R2C2 model in residency education: how does it foster coaching and promote feedback use?. Acad Med.

[16] Sargeant J, Mann K, Manos S (2017). R2C2 in action: testing an evidence-based model to facilitate feedback and coaching in residency. J Grad Med Educ.

[17] Norris TE, Schaad DC, DeWitt D, Ogur B, Hunt DD, MBA members of the Consortium of Longitudinal Integrated Clerkships (2009). Longitudinal integrated clerkships for medical students: an innovation adopted by medical schools in Australia, Canada, South Africa, and the United States. Acad Med.

[18] Karp NC, Hauer KE, Sheu L (2019). Trusted to learn: a qualitative study of clerkship students’ perspectives on trust in the clinical learning environment. J Gen Intern Med.

[19] Sklar DP, McMahon GT (2019). Trust between teachers and learners. JAMA.

[20] Holzhausen Y, Maaz A, Cianciolo AT, ten Cate O, Peters H (2017). Applying occupational and organizational psychology theory to entrustment decision-making about trainees in health care: a conceptual model. Perspect Med Educ.

[21] ten Cate O, Hart D, Ankel F (2016). Entrustment decision making in clinical training. Acad Med.

[22] American Medical Association. Coaching in medical education: guidance for educators and students. 2020. https://www.ama-assn.org/education/accelerating-change-medical-education/coaching-medical-education-guidance-educators-and. Accessed 12 Apr 2020.

[23] Irby DM, Cooke M, O’Brien BC (2010). Calls for reform of medical education by the Carnegie Foundation for the Advancement of Teaching: 1910 and 2010. Acad Med.

[24] Cutrer WB, Atkinson HG, Friedman E (2018). Exploring the characteristics and context that allow master adaptive learners to thrive. Med Teach.

[25] Cutrer WB, Miller B, Pusic MV (2017). Fostering the development of master adaptive learners: a conceptual model to guide skill acquisition in medical education. Acad Med.

[26] Lajoie SP, Gube M (2018). Adaptive expertise in medical education: accelerating learning trajectories by fostering self-regulated learning. Med Teach.

[27] Artino ARJ, Dong T, DeZee KJ (2012). Achievement goal structures and self-regulated learning: relationships and changes in medical school. Acad Med.

[28] Berkhout JJ, Helmich E, Teunissen PW, van den Berg JW, van der Vleuten CPM, Jaarsma ADC (2015). Exploring the factors influencing clinical students’ self-regulated learning. Med Educ.

[29] Berkhout JJ, Helmich E, Teunissen PW, van der Vleuten CPM, Jaarsma ADC (2017). How clinical medical students perceive others to influence their self-regulated learning. Med Educ.

[30] Cho KK, Marjadi B, Langendyk V, Hu W (2017). Medical student changes in self-regulated learning during the transition to the clinical environment. BMC Med Educ.

[31] Murad MH, Coto-Yglesias F, Varkey P, Prokop LJ, Murad AL (2010). The effectiveness of self-directed learning in health professions education: a systematic review. Med Educ.

[32] Ahmed K, Ashrafian H (2009). Life-long learning for physicians. Science.

[33] Lawlor KB. Smart goals: how the application of smart goals can contribute to achievement of student learning outcomes. 2012. https://absel-ojs-ttu.tdl.org/absel/index.php/absel/article/view/90. Accessed 12 Apr 2020, Dev Bus Simul Exp Learn Proc Annu ABSEL Conf.

[34] Jarvis-Selinger S, Pratt DD, Regehr G (2012). Competency is not enough: integrating identity formation into the medical education discourse. Acad Med.

[35] Cruess RL, Cruess SR, Boudreau JD, Snell L, Steinert Y (2014). Reframing medical education to support professional identity formation. Acad Med.

[36] Sternszus R, Boudreau JD, Cruess RL, Cruess SR, Macdonald ME, Steinert Y. Clinical teachers’ perceptions of their role in professional identity formation. 2020. https://journals.lww.com/academicmedicine/Abstract/publishahead/Clinical_Teachers__Perceptions_of_Their_Role_in.97230.aspx. Accessed 12 Apr 2020, Acad Med. 10.1097/ACM.0000000000003369 32271232

[37] Niemi PM, Vainiomaki PT, Murto-Kangas M (2003). “My future as a physician”-professional representations and their background among first-day medical students. Teach Learn Med.

[38] Wong A, Trollope-Kumar K (2014). Reflections: an inquiry into medical students’ professional identity formation. Med Educ.

[39] Weaver R, Peters K, Koch J, Wilson I (2011). ‘Part of the team’: professional identity and social exclusivity in medical students. Med Educ.

[40] Watling CJ, LaDonna KA (2019). Where philosophy meets culture: exploring how coaches conceptualise their roles. Med Educ.

[41] Hauer KE, O’Brien BC, Hansen LA (2012). More is better: students describe successful and unsuccessful experiences with teachers differently in brief and longitudinal relationships. Acad Med.

[42] UVA Today. Teaching wisdom: Phronesis project brings practical wisdom to medical school. 2016. https://news.virginia.edu/content/teaching-wisdom-phronesis-project-brings-practical-wisdom-medical-school. Accessed 13 Apr 2020.

[43] Molloy E, Bearman M (2019). Embracing the tension between vulnerability and credibility: ‘intellectual candour’ in health professions education. Med Educ.

[44] Plack MM, Driscoll M, Marquez M, Greenberg L (2010). Peer-facilitated virtual action learning: reflecting on critical incidents during a pediatric clerkship. Acad Pediatr.

[45] ten Cate O, Chen HC, Hoff RG, Peters H, Bok H, van der Schaaf M (2015). Curriculum development for the workplace using Entrustable Professional Activities (EPAs): AMEE Guide No. 99. Med Teach.

[46] Keeley MG, Gusic ME, Morgan HK, Aagaard EM, Santen SA (2019). Moving toward summative competency assessment to individualize the postclerkship phase. Acad Med.

[47] Lockspeiser TM, Kaul P (2016). Using individualized learning plans to facilitate learner-centered teaching. J Pediatr Adolesc Gynecol.

[48] Li S-TT, Tancredi DJ, Co JPT, West DC (2010). Factors associated with successful self-directed learning using individualized learning plans during pediatric residency. Acad Pediatr.

[49] Farrell L, Bourgeois-Law G, Buydens S, Regehr G (2019). Your goals, my goals, our goals: the complexity of coconstructing goals with learners in medical education. Teach Learn Med.

